# Effects of crocin and zinc chloride on blood levels of zinc and metabolic and oxidative parameters in streptozotocin-induced diabetic rats

**Published:** 2015

**Authors:** Siamak Asri-Rezaei, Esmaeal Tamaddonfard, Behnaz Ghasemsoltani-Momtaz, Amir Erfanparast, Sima Gholamalipour

**Affiliations:** 1 ^*‎*^*Division of Clinical Pathology, Department of Clinical Sciences, Faculty of Veterinary Medicine, Urmia University, Urmia, Iran*; 2 *Division of Physiology, Department of Basic Sciences, Faculty of Veterinary Medicine, Urmia University, Urmia, Iran*

**Keywords:** *Crocin*, *Rats*, *Diabets*, *Zinc*

## Abstract

**Objectives:**

Crocin is one of constituents of saffron and has antioxidant property. Zinc chloride is one of the common compounds of zinc with antioxidant activity. The present study was aimed to investigate separate and combined treatment effects of crocin and zinc chloride on blood levels of zinc and metabolic and oxidative parameters in diabetic rats.

**Materials and Methods:**

Diabetes was induced by intraperitoneal (i.p.) injection of 50 mg/kg of streptozotocin (STZ) and was confirmed by blood glucose levels higher than 250 mg/dL. After confirmation of diabetes, injections (i.p.) of crocin and zinc chloride were performed for six weeks. At the end of the experiment, blood levels of zinc, glucose, insulin, malodialdehyde (MDA), and total antioxidant capacity (TAC) were measured. ‎

**Results:**

Crocin (25 and 50 mg/kg) and zinc chloride (5 mg/kg) significantly recovered the decreased levels of zinc, insulin, and TAC and improved the increased levels of glucose and MDA in STZ-induced diabetic rats. In a combination treatment performed with an ineffective dose of crocin (12.5 mg/kg) and a low dose of zinc chloride (1.25 mg/kg), improving effects were observed on the above-mentioned biochemical parameters.‎

**Conclusion:**

The results indicated that separate and combined treatments with crocin and zinc chloride produced improving effects on the blood levels of zinc, glucose, insulin, MDA and TAC in STZ-induced diabetic rats.

## Introduction

The prevalence of diabetes across all age groups worldwide was estimated to be 2.8% in 2000 and will rise to 4.4% in 2030. Total number of people with diabetes is projected to rise from 171 million in 2000 to 366 million in 2030 (Wild et al., 2004 ▶). Diabetes mellitus type 1 is a chronic autoimmune disorder caused by lymphocytic infiltration and β-cell destruction within the pancreatic islets of Langerhans. The pancreatic β-cells are lost in number and volume leading to severe permanent insulin deficiency and chronic hyperglycemia (Bluestone et al., 2010 ▶). Chronic hyperglycemia causes increased production of free radicals for all tissues from glucose auto-oxidation and protein-glycosylation. The chronic hyperglycemia and the generation of reactive oxygen species (ROS) are strong predictors of the development of diabetic complications (Niedowicz and Daleke, 2005 ▶). 

Zinc is an intracellular signaling molecule and plays important roles in growth and development, sensory function, blood clotting, reproduction, and immune system (Prasad, 2009 ▶; Tudor et al., 2005 ▶). It also has potent antioxidant and anti-inflammatory properties (Prasad, 2009 ▶). Zinc has been shown to exert anti-diabetic effects in various experimental models and in a limited number of human studies (Vardatsikos et al., 2013 ▶). In this context, Abou-Seif and Youssef (2004) ▶ reported a decreased level of plasma zinc in diabetic patients. Moreover, serum levels of zinc were decreased after induction of diabetes with twice STZ injection in rats and supplementation with zinc recovered this effect (Bicer et al., 2011 ▶). 

Crocin [digentiobiosyl all-*tarns*-crocetin (8,8′-di-apocarotene-8,8′-dioic acid) ester] is one of the active substances of saffron and gardenia yellow, the extracts of *Crocus sativus* stigmasand* Gardenia jasminoides* fruits, respectively (Hosseinzadeh and Nassiri-Asl, 2013 ▶; Lee et al., 2005 ▶). Pharmacological studies have suggested anti–oxidant and anti–inflammatory (Poma et al., 2012 ▶; Tamaddonfard et al., 2012a ▶), antinociceptive (Tamaddonfard and Hamzeh-Gooshci, 2010a ▶, 2010b ▶; Tamaddonfard et al., 2013c ▶; Karami et al., 2013 ▶), neuroprotective (Tamaddonfard et al., 2013a ▶, 2013b ▶), antiepileptic (Tamaddonafrd et al., 2012b ▶), and anticancer (Zhang et al., 2013 ▶) properties for crocin. Recent studies have shown improving effects for crocin in both type 1 and type 2 diabetes (Shirali et al., 2013 ▶; Rajaei et al., 2013 ▶; Tamaddonfard et al., 2013b ▶).

The etiology of diabetes and its complications are complex, and prevention and treatment require combined use of anti-diabetic drugs, insulin, vitamins, trace elements, and herbal preparations (Triggiani et al., 2006 ▶). In this context, medicinal plants and their active constituents have received considerable attention for the management of diabetes and its complications (Patel et al., 2012a ▶, 2012b ▶). In the present study, we investigated separate and combined treatment effects of crocin and ZnCl_2_ on blood levels of zinc, glucose, insulin, malodialdehyde (MDA), and total antioxidant capacity (TAC) in streptozotocin (STZ)-induced type 1 diabetic rats.

## Materials and Methods

**Animals**

Healthy adult male Wistar rats, weighing 210–250 g were used in this study. Rats were maintained in polyethylene cages with food and water available *ad libitum* in a laboratory with controlled ambient temperature (22–23°C) and under a 12 h light-dark cycle (lights on 07:00 h). Six rats were used in each experiment. All research and animal care procedures were approved by the Veterinary Ethics Committee of the Faculty of Veterinary Medicine of Urmia University and were performed in accordance with the National Institutes of Health Guide for Care and Use of Laboratory Animals.

**Drugs and chemicals**

Crocin and zinc chloride were purchased from Sigma-Aldrich (St. Louis, MO, USA). The chemicals were dissolved in sterile normal saline. Glucose test kit was obtained from Zistshimi, Tehran, Iran. Insulin ELISA test kit was obtained from DRG instruments GmbH, Marburg, Germany, Cat no. (EIA 2935). All of the analytical chemicals used in the present study were purchased from Merck Chemical Co. (Darmstadt, Germany).

**Treatment groups**

In the present study, 48 male Wistar rats were divided into eight groups each containing six rats. Group 1 received citrate buffer followed by normal saline. Group 2 received STZ followed by normal saline. Groups 3, 4, and 5 received STZ followed by 12.5, 25, and 50 mg/kg of crocin, respectively. Groups 6 and 7 received STZ followed by 1.25 and 5 mg/kg of zinc chloride. Group 8 received STZ followed by 12.5 mg/kg of crocin plus 1.25 mg/kg of zinc chloride. Crocin and zinc chloride were injected (i.p.) at volumes of 1 ml/kg five days weekly for six weeks. In the present study, the doses of crocin and zinc chloride were designed according to previous studies in which crocin (15, 30, and 60 mg/kg for six weeks) and zinc chloride (5 mg/kg for 1 month) were used (Rajaei et al., 2013 ▶; Aly and Mantawy, 2012 ▶). 

**Induction of diabetes**

Diabetes was induced in overnight fasted rats by a single injection (i.p.) of 50 mg/kg of freshly prepared STZ. STZ (2-deoxy-2(-3-(methyl-3-nitrosoureido-D-glucopyranose) is synthesized by *Streptomycetesachromogenes* and is used to induce both insulin-dependent and non-insulin-dependent diabetes mellitus (King, 2012 ▶; Islam and Loots, 2009 ▶). The frequently used single intravenous dose of STZ in adult rats to induce type 1 diabetes is between 40 and 60 mg/kg, but higher doses are also used. STZ is also efficacious after injection (i.p.) of a similar or high dose, but a single dose below 40 mg/kg may be ineffective (Szkudelski, 2001 ▶). The STZ was dissolved in sodium citrate buffer (0.1 M, pH 4.5). Hyperglycemia was confirmed by the elevated glucose levels in plasma of 12–h fasted rats, determined on day 3 after injection of STZ, using a digital glucometer (Elegans, Germany).

**Blood sampling**

At the end of the experiments, the animals were deeply anaesthetized with injection (i.p.) of a mixture of ketamine (150 mg/kg) and xylazine (15 mg/kg). A 23-gauge injection needle was inserted into the heart through seventh and eighth intercostals muscles (Farshid et al., 2013 ▶) and blood samples were collected into non-coagulant and heparin-containing tubes to obtain plasma and serum samples. Plasma and serum samples were kept at –80°C until analysis.

**Biochemical assay**

Zinc concentration was measured in duplicate after 20-fold dilution of serum in double distilled water by flame atomic absorption spectrophotometer (Schimadzu, AA6800, Tokyo, Japan) at 213.9 nm. The standards were prepared from a 1 mg/ml zinc nitrate using 5% glycerol to approximate the viscosity characteristics and to avoid any contamination from exogenous sources. All tubes were soaked in HCl (10% v/v) for 16 h and rinsed with double distilled water (Kiilerich et al., 1980 ▶). Serum levels of zinc were expressed as μg/dL. Blood glucose was measured using enzymatic colorimetric–GOD–PAP (glucose oxidase-phenol-4-aminoantipyrine) method. Blood glucose levels were expressed as mg/dL.

Serum insulin concentration was detected using an ELISA test kit after serum samples were thawed at room temperature. Serum insulin levels were expressed as IU/ml.

Plasma MDA levels were measured by the thiobarbitoric (TBA) acid method that was modified from method of Yagi, (1984) ▶. Peroxidation was measured as the production of MDA, which in combination with TBA forms a pink chromogen compound whose absorbance was measured spectrophotometrically (JASCO, UV–975, Tokyo, Japan) at 532 nm. Plasma MDA results were expressed as nmol/ml.

Plasma TAC was determined by measuring the ability to reduce Fe^3+^ to Fe^2+^ as named ferric-reducing antioxidant power (FRAP) as described by Benzie and Strain (1996) ▶. The reagent included TPTZ (2,4,6-tripiyryidyl-s-triazine), FeCl_3_, and acetate buffer. Twenty microliter of water-diluted plasma was added to 600 microliter of freshly prepared reagent warmed at 37°C. The complex between Fe^2+^ and TPTZ gives a blue color with absorbance at 593 nm. Plasma TAC levels were expressed as nmol/ml.

**Statistical analysis**

Differences between groups were assessed by one-way analysis of variance (ANOVA) followed by Duncan’s test. The values are expressed as mean ± SEM for six animals. Significance at p<0.05 has been given receptive in the figures.

## Results

**Figure 1:**
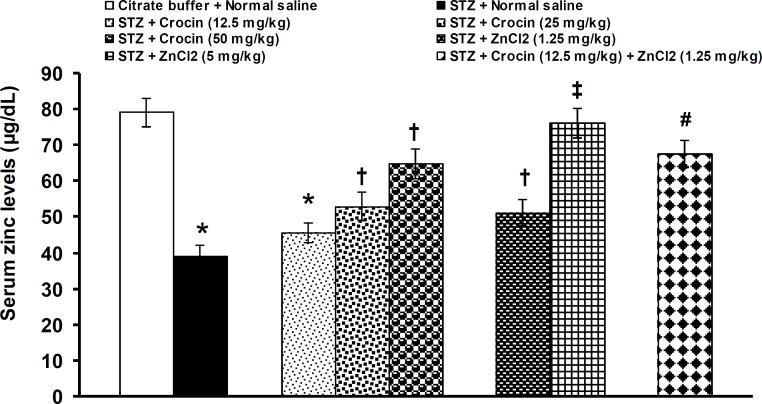
Effects of separate and combined treatments with crocin and zinc chloride on serum levels of zinc in STZ-induced diabetic rats. All values are expressed as mean ± SEM (n = 6). *p<0.05 in comparison with citrate buffer-treated group.^†^p<0.05 in comparison with citrate buffer- and STZ-treated groups.^‡^p<0.05 in comparison with STZ-treated group.^#^p<0.05 in comparison with citrate- buffer-, STZ-, crocin- (12.5 mg/kg), and zinc chloride- (1.25 mg/kg) treated groups. STZ: streptozotocin, ZnCl_2_: zinc chloride

STZ significantly (p<0.05) decreased serum levels of zinc (STZ: 39.3 ± 2.8 μg/dL*vs.* normal saline: 79.2 ± 4.0μg/dL).Crocin at a dose of 12.5 did not alter the decreased levels of blood zinc. Crocin at doses of 25 and 50 mg/kg and zinc chloride at doses of 1.25 and 5 mg/kg significantly (p<0.05) increased the decreased levels of zinc. An ineffective dose of crocin (12.5 mg/kg) significantly (p<0.05) increased the improving effect of 1.25 mg/kg of zinc chloride on zinc levels when used together (Figure1). Blood glucose levels were significantly (p<0.05) increased by STZ (STZ: 480.8 ± 41.8 mg/dL*vs.* normal saline: 129.3±10.4 mg/dL). Crocin at a dose of 12.5 did not change the increased levels of blood glucose. 

**Figure 2:**
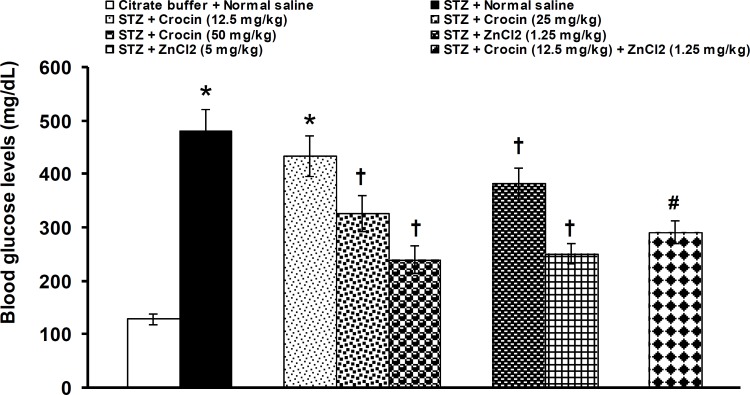
Effects of separate and combined treatments with crocin and zinc chloride on blood levels of glucose in STZ-induced diabetic rats. All values are expressed as mean ± SEM (n = 6). *p<0.05 in comparison with citrate buffer-treated group.^†^p<0.05 in comparison with STZ-treated group.^#^p<0.05 in comparison with citrate buffer-, STZ-, crocin- (12.5 mg/kg), and zinc chloride- (1.25 mg/kg) treated groups. STZ: streptozotocin, ZnCl_2_: zinc chloride

**Figure 3:**
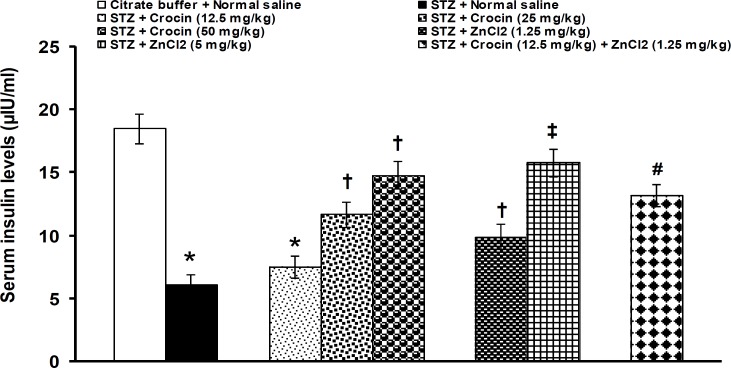
Effects of separate and combined treatments with crocin and zinc chloride on serum levels of insulin in STZ-induced diabetic rats. All values are expressed as mean ± SEM (n = 6). *p<0.05 in comparison with citrate buffer-treated group.^†^p<0.05 in comparison with citrate buffer- and STZ-treated groups.^‡^p<0.05 in comparison with STZ-treated group.^#^p<0.05 in comparison with citrate buffer-, STZ-, crocin- (12.5 mg/kg), and zinc chloride- (1.25 mg/kg) treated groups. STZ: streptozotocin, ZnCl_2_: zinc chloride

Crocin at doses of 25 and 50 mg/kg and zinc chloride at doses of 1.25 and 5 mg/kg significantly (p<0.05) decreased the increased levels of glucose. When an ineffective dose of crocin (12.5 mg/kg) was concurrently used with an effective dose of zinc chloride (1.25 mg/kg), the reduction of blood glucose levels was documented (Figure 2).Serum insulin levels were significantly (p<0.05) decreased by STZ (STZ: 6.1 ± 0.8 μIU/ml *vs.* normal saline 18.5 ± 1.19 μIU/ml). Crocin at a dose of 12.5 did not change the increased levels of blood insulin. Crocin at doses of 25 and 50 mg/kg and zinc chloride at doses of 1.25 and 5 mg/kg significantly (p<0.05) elevated the reduced levels of insulin. An ineffective dose of crocin (12.5 mg/kg) significantly (p<0.05) increased the improving effect of 1.25 mg/kg of zinc chloride on insulin levels when used together (Figure 3).

**Figure 4:**
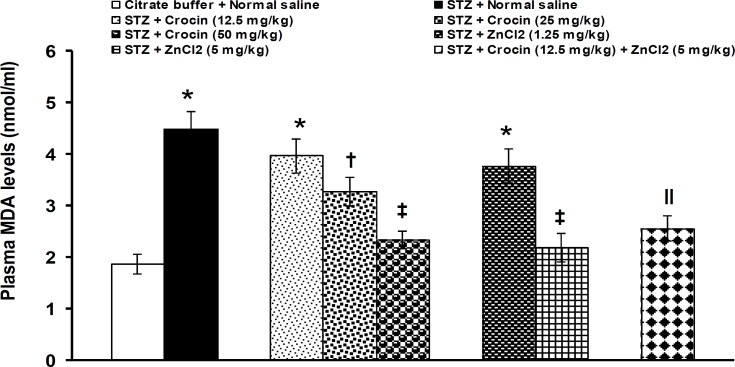
Effects of separate and combined treatments with crocin and zinc chloride on plasma levels of MDA in STZ-induced diabetic rats. All values are expressed as mean ± SEM (n = 6). *p<0.05 in comparison with citrate buffer-treated group.^†^p<0.05 in comparison with STZ-treated group.^‖^p<0.05 in comparison with STZ-, crocin- (12.5 mg/kg), and zinc chloride- (1.25 mg/kg) treated groups. STZ: streptozotocin, MDA: malodialdehyde, ZnCl_2_: zinc chloride

Plasma levels of MDA were significantly (p<0.05) increased by STZ (STZ: 4.48 ± 0.36 nmol/ml *vs* normal saline: 1.88 ± 0.19 nmol/ml). Crocin and zinc chloride at doses of 12.5 mg/kg and 1.25 mg/kg, respectively, did not alter the increased levels of blood MDA. Crocin at doses of 25 and 50 mg/kg and zinc chloride at a dose of 5 mg/kg significantly (p<0.05) improved the changed levels of MDA. Improving effects on blood levels of MDA were observed when ineffective doses of crocin (12.5 mg/kg) and zinc chloride (1.25 mg/kg) were used together (Figure 4).

**Figure 5:**
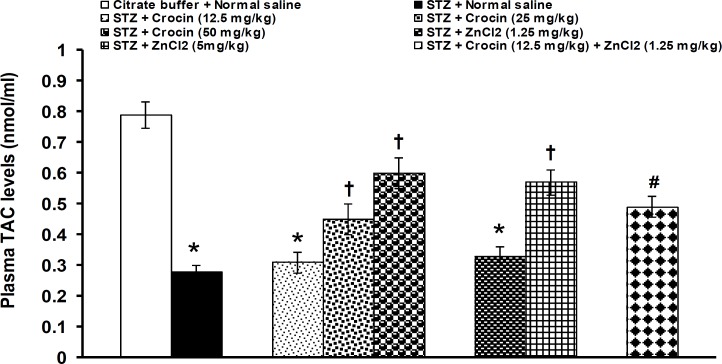
Effects of separate and combined treatments with crocin and zinc chloride on plasma levels of total antioxidant capacity (TAC) in STZ-induced diabetic rats. All values are expressed as mean ± SEM (n = 6). *p<0.05 in comparison with citrate buffer-treated group.^†^p<0.05 in comparison with STZ-treated group.^#^p<0.05 in comparison with citrate buffer-, STZ-, crocin- (12.5 mg/kg) and zinc chloride- (1.25 mg/kg) treated groups. STZ: streptozotocin, ZnCl_2_: zinc chloride

Plasma levels of TAC were significantly (p<0.05) decreased by STZ (STZ: 0.28 ± 0.02 nmol/ml *vs* normal saline: 0.79 ± 0.04 nmol/ml). Crocin and zinc chloride at doses of 12.5 mg/kg and 1.25 mg/kg, respectively, did not change the decreased levels of blood TAC. Crocin at doses of 25 and 50 mg/kg and zinc chloride at a dose of 5 mg/kg significantly (p<0.05) increased the decreased levels of TAC. When ineffective doses of crocin (12.5 mg/kg) and zinc chloride (1.25 mg/kg) were used together, an improving effect was observed on blood levels of TAC (Figure 5).

## Discussion

The results of the present study showed that STZ decreased the blood levels of zinc. Zinc is the second most abundant trace element in the human body and is important nutrient and cofactor of numerous enzymes and transcription factors (Vallee and Falchuk, 1993 ▶). A general observation in diabetes, type 1 as well as type 2, is a loss of zinc, and the resulting decrease in total body zinc may contribute to diabetic complications (Chausmer, 1998 ▶; Miao et al., 2013 ▶; Vardatsikos et al., 2013 ▶). In young people with type 1 diabetes mellitus, Lin et al., (2014) ▶ found lower levels of serum zinc.

Moreover, Navarro-Alarcan et al., 2013 ▶ showed a decreased level of plasma zinc in Zucker diabetic rats. Beside blood, a reduction of zinc was reported in kidney and liver of diabetic rats (Ozcelik et al., 2011 ▶).

Increasing evidence indicates that diabetes can affect zinc homeostasis in many ways, but it is most likely the hyperglycemia rather than any primary lesion related to diabetes, to cause the increased urinary loss and decrease in total body zinc (Miao et al., 2013 ▶; Vardatsikos et al., 2013 ▶). In the present study, crocin and zinc chloride increased the decreased levels of blood zinc. There are not any reports showing the effects of crocin on serum levels of zinc in diabetic rats. In STZ-induced diabetic rats, oral administration of *Jasadabhasma* (zinc ash) improved the decreased serum levels of zinc (Umrani et al., 2013 ▶). Bicer et al., (2011) ▶ reported that zinc sulfate (6 mg/kg, i.p., 1 month) supplementation increased the decreased levels of plasma zinc in STZ-induced diabetic rats. Although the exact mechanisms by which zinc supplementation prevents diabetic complications remains unknown, several possibilities including insulin-like effect and antioxidant property of zinc have been implicated (Miao et al., 2013 ▶; Vardatsikos et al., 2013 ▶). 

In the present study, STZ produced hyperglycemia and hypoinsulinemia. These results are in agreement with other investigations (Shirali et al., 2013 ▶; Rajaeiet al., 2013 ▶; Tamaddonfard et al., 2013b ▶). In our study, crocin and zinc chloride improved the hyperglycemia and hypoinsulinemia induced by STZ. Shirali et al., 2013 ▶ reported that crocin decreased serum glucose and microalbuminuria and reduced insulin sensitivity in STZ-induced type 2 diabetic rats. In addition, crocin reduced the blood glucose level and lipid peroxidation levels in kidney and liver of STZ-induced type 1 diabetic rats (Rajaei et al., 2013 ▶). In STZ-induced type 1 diabetic rats, long term (30 days) administration of crocin recovered blood glucose and insulin levels (Tamaddonfard et al., 2013b ▶). Treatment of diabetic rats with zinc chloride improved the increased serum levels of glucose (Aly and Mantawy, 2012 ▶). In STZ-induced diabetic rats, oral administration of zinc threoninate chelate (3, 6 and 9 mg/kg for 7 weeks) reduced blood levels of glucose and increased serum levels of insulin (Zhu et al., 2013 ▶). Zinc plays a key role in the synthesis and action of insulin, both physiologically and in the pathological state of diabetes (Chausmer, 1998 ▶). At the level of pancreatic beta-cells, zinc is essential for the correct processing, storage, secretion and action of insulin (Li, 2014 ▶).

In the present study, STZ increased and decreased plasma levels of MDA and TAC, respectively. Treatments with crocin and zinc chloride recovered these effects of STZ. Living organisms have developed complex antioxidant systems to counteract reactive species and to reduce their damage. These antioxidant systems include enzymes such as superoxide dismutase, macromolecules such as ceruloplasmin, and an array of small molecules including ascorbic acid, β-carotene, uric acid and bilirubin (Yu, 1994 ▶). MDA, an end product of polyunsaturated fatty acid, is a reliable and commonly used biomarker for assessing lipid peroxidation. Lipid peroxidation is a well-established mechanism of cellular injury and is used as an indicator of oxidative stress in cells and tissues (Moore and Roberts 1998 ▶). In STZ-induced diabetic rats, the increased levels of plasma MDA and the decreased levels of TAC and recovering effect of crocin on plasma changes of MDA and TAC were reported (Tamaddonfard et al, 2013b ▶). Treatment of diabetic rats with zinc chloride improved recovered the liver levels of nitric oxide, and superoxide dismutase (SOD), lactate dehydrogenase (LDH), pyruvate kinase (PK) and MDA (Aly and Mantawy, 2012 ▶). In STZ-induced diabetic rats, oral administration of zinc threoninate chelate decreased plasma levels of MDA and improved antioxidant enzymes activity (Zhu et al., 2013 ▶). It is well known that zinc is an important component of the body’s antioxidant system and plays a role in retarding the oxidative processes particularly related to diabetes mellitus (DiSilvestro 2000 ▶).

In the present study, an ineffective dose of crocin enhanced the improving effects of zinc on blood levels of zinc, glucose and insulin. Moreover, ineffective doses of crocin and zinc produced improving effects on blood levels of MDA and TAC. Although there is not any report showing the interaction between crocin and zinc, some interactions have been reported between zinc and other antioxidant agents. It has been reported that low serum levels of vitamin C and zinc may associate with an increased risk for the development of oxidative stress in type 2 diabetes mellitus with periodontitis (Pushparani et al., 2013 ▶). In addition, Aly and Mantawy (2012) ▶ suggested a beneficial effect of vitamin E and zinc combination in controlling hyperglycemia in STZ-induced diabetic rats. On the other hand, some researchers have reported some interactions between crocin with drugs such as morphine and cyclooxygenase inhibitors (Tamaddonfard and Hamzeh-Gooshchi, 2010a ▶, 2010b ▶; Tamaddonfard et al., 2013c ▶).

In conclusion, the results of the present study showed that crocin and zinc chloride improved the decreased levels of zinc in STZ-induced diabetic rats, and produced anti-hyperglycemic, anti-hypoinsulinemic and antioxidant activities. 
